# Cerebrovascular disease in patients with COVID-19 infection: a case series from Lebanon

**DOI:** 10.1097/MS9.0000000000000953

**Published:** 2023-06-10

**Authors:** Zeinab El Mawla, Ghaidaa El Saddik, Maya Zeineddine, Mahmoud Hassoun, Taghrid El Hajj

**Affiliations:** a Faculty of Medicine, Department of Pulmonary & Critical Care; b Faculty of Medicine, Lebanese University; c Faculty of Medicine, Department of Internal Medicine, Beirut Arab University; d Middle East and North Committee for Treatment and Research in Multiple Sclerosis (MENACTRIMS), Beirut; e Department of Pulmonary & Critical Care, Rafic Hariri University Hospital, Babda, Lebanon

**Keywords:** stroke, ischaemic, haemorrhagic, covid-19, Lebanon, neurologic complications

## Abstract

COVID-19 has been associated with a variety of multi-organs complications, with an increasing proportion of patients presenting with neurologic manifestations. There is still an uncertainty in the relationship between stroke and COVID-19. Therefore, in this study, the authors report 18 cases of acute stroke occurring in the setting of COVID-19 infection, including 11 ischaemic strokes and 7 haemorrhagic strokes and identified in a Lebanese tertiary hospital. In this case series, patients with ischaemic and haemorrhagic stroke had elevated markers of inflammation and coagulation. Ischaemic stroke patients were treated with different regimens of anti-platelets, anticoagulants, and thrombolytic therapies. Death was the most common outcome observed and was associated with the severity of COVID-19 infection.

## Introduction

Highlights

- Only required for full research articles.
- It is a case-series study.

Since 22 January 2020, the worldwide number of confirmed cases of COVID-19 reached over 350 million with a resultant mortality of over 5.6 million as reported to the WHO^1^. While primarily a respiratory disease, COVID-19 has been associated with a variety of multi-organs complications, with an increasing proportion of patients presenting with neurologic manifestations^2^. Neurologic sequelae have been reported in a number of COVID-19 patients including ischaemic and haemorrhagic strokes, seizures, Guillain–Barre Syndrome, encephalopathy, and encephalitis^3^.

The clinical presentation of COVID-19 varies between asymptomatic infection, mild respiratory infection, severe pneumonia with respiratory failure and death^4^. Raised neutrophil-lymphocyte ratio, mean platelet volume, interleukin-6 (IL-6), D-dimer, serum ferritin, lactate dehydrogenase, and C-reactive protein (CRP) were important prognostic markers in determining the severity of COVID-19^5^. Zhou *et al*.^6^ showed that older age, higher d-dimer levels, and higher Sequential Organ Failure Assessment (SOFA) scores in COVID-19 patients at admission were associated with high in-hospital mortality. In addition, increased levels of cTnI, lactate dehydrogenase, and lymphocytopenia were more common in patients with severe COVID-19. Moreover, studies have shown that SARS-CoV-2 can participate in and induce the activation of the complement and coagulation system, which is related to the severity of COVID-19 patients^7^. Moreover, COVID-19 has been associated with a hypercoagulable state causing cardiovascular and neurological complications^8^. Postmortem histology of all organs studied in patients infected with SARS-CoV-2 demonstrated macrovascular and microvascular thrombosis in the arterioles, capillary bed, and venules consisting of platelets, fibrin, red blood cells, and leucocytes, supporting the fact that, rather than merely infecting the airway, SARS-CoV-2 induces a disease in the vasculature^9^. Evidence is mounting on the diverse neurological presentations associated with COVID-19. The reported incidence of cerebrovascular disease in patients testing positive for SARS-CoV-2 ranges from 1 to 6%, potentially equating to large numbers of individuals as the pandemic progresses in some countries^10^.

Multiple risk factors associated with SARS-CoV-2 and the development of COVID-19 could contribute to the increased severity and earlier onset of acute ischaemic stroke including generalized hypercoagulability, dysregulated immune response leading to cytokine-release syndrome, damage to endothelial cells leading to increased inflammation and thrombosis, dysregulation of the renin-angiotensin-aldosterone system, direct cytotoxic effect on the nervous system related to angiotensin converting enzyme-2 receptor uptake of SARS-CoV-2 virus, hypoxaemia related to cardiorespiratory failure, and metabolic derangement^9^.

Because the treatment of ischaemic strokes includes different regimens of anti-platelets, anticoagulants, and thrombolytic therapies; it is important to understand the relation between COVID-19 and coagulative changes in the body. So far, a lot about the virus and its behaviour is unknown^11^.

For a better characterization of cerebrovascular complications associated with COVID-19 infection, we herein report the clinical presentation, neuroimaging findings, and outcome of eighteen patients developing acute stroke during COVID-19 infection.

## Methods

After the announcement of the outbreak of COVID-19, a public hospital became one of the leading referral centres for COVID-19 on February 2020 in Lebanon.

A single-centre retrospective chart review of all hospitalized cases with confirmed COVID-19 infection (SARS-CoV-2 real-time reverse transcription-polymerase chain reaction positive) and cerebrovascular diseases (ischaemic and haemorrhagic stroke) between 1 November 2020 and 30 June 2021 was performed.

About 1817 patients were admitted with moderate to severe COVID-19 diagnosis, amongst which 18 had ischaemic or haemorrhagic stroke. According to hospital guidelines, the diagnosis of COVID-19 infection was made through clinical history, positive COVID-19 PCR, and chest computed tomography (CT) scan. All COVID-19 patients with stroke symptoms underwent a brain CT scan, with or without CT angiography of brain vessels and were monitored continuously for blood oxygen saturation and need for mechanical ventilation.

Clinical data were obtained from inpatient medical charts. Blood and imaging results were extracted from the electronic medical records. The Modified Rankin Scale (mRS) was used to determine the disability upon discharge which varies from 0 (no symptoms) to 6 (death). For ischaemic strokes, the trial of ORG 10172 in acute stroke treatment (TOAST) classification was either extracted from the discharge summary or was inferred from the clinical team’s documented assessment of likely stroke aetiology. This study was approved by the Institutional Review Board (IRB) as exempted.

The work has been reported in line with the PROCESS criteria^12^.

## Results

**Figure 1:**
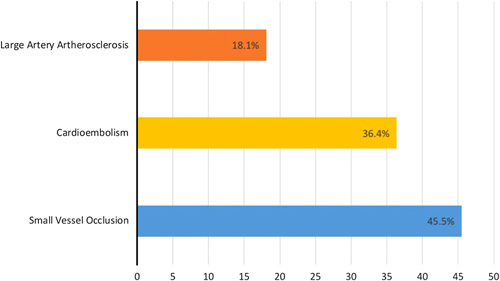
Classification of ischaemic stroke according to TOAST^*^ criteria (*n*=11). *TOAST Criteria = The TOAST (trial of ORG 10172 in acute stroke treatment) classification denotes five sub types of ischaemic stroke: large-artery atherosclerosis (embolus / thrombosis); cardioembolism (high-risk / medium-risk); small-vessel occlusion (lacune); stroke of other determined aetiology; stroke of undetermined aetiology. *X*-axis showed the percentage of patients with different types of ischaemic stroke.

**Figure 2:**
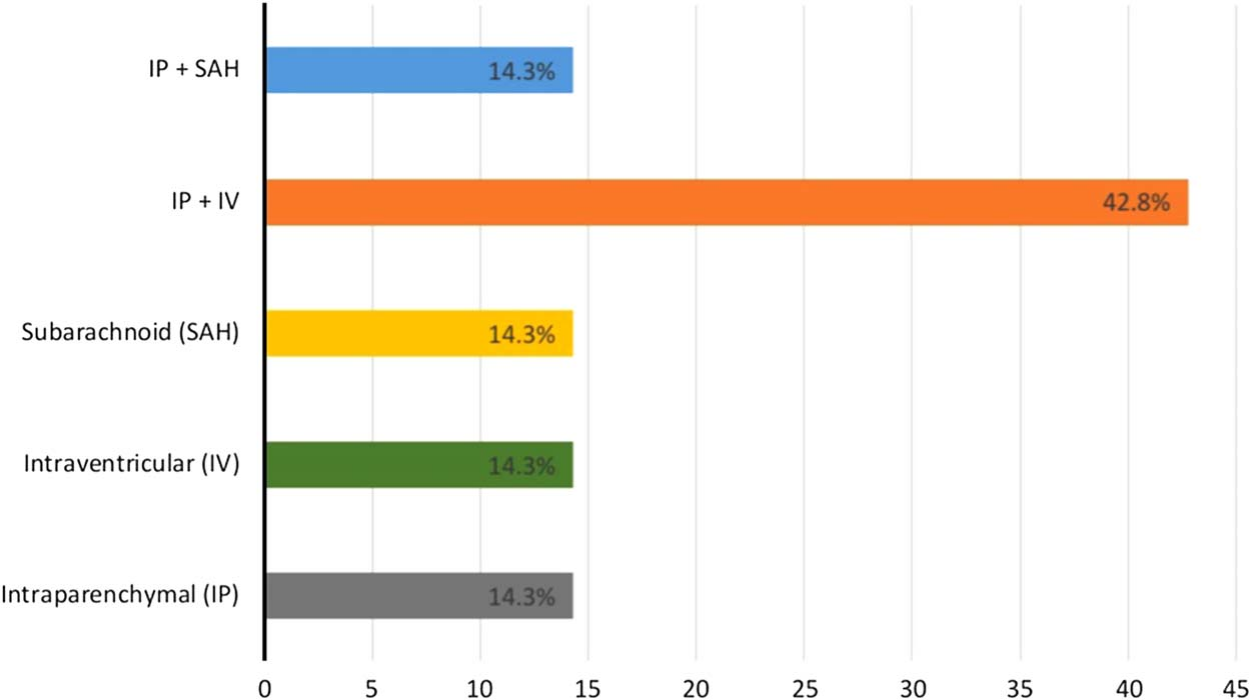
Distribution of intracerebral haemorrhage (*n*=7). *X*-axis showed the percentage of patients with different types of haemorrhagic stroke. IP, intraparenchymal; IV, intraventricular; SAH, subarachnoid haemorrhage.

We report on18 patients with COVID-19 and concurrent strokes:11 (61.1%) with ischaemic and 7 (38.9%) with haemorrhagic strokes. Ten (55.6%) of the patients were males. Baseline demographics, clinical and neurological characteristics, and laboratory parameters of patients with COVID-19 infection who developed stroke are presented in Table 1. The most common comorbidities were hypertension (83.3%) and diabetes mellitus (58.3%). The majority of our patients (77.8%) had respiratory symptoms as their initial COVID-19 symptoms. The most common stroke symptoms were hemiparesis (44.4%), followed by coma (38.9%). Based on the Toast criteria for classification of ischaemic stroke, four patients had cardio-embolic aetiology of their stroke, four patients had large-artery atherosclerosis, and three patients had a stroke of undetermined aetiology (Fig. 1). In patients with haemorrhagic stroke, the most common location was a combination of intraparenchymal and intraventricular haemorrhage (Fig. 2). The neuroimaging characteristics of patients are tabulated in Table 2.

**Table 1:** Baseline demographics, clinical and neurological characteristics of patients with confirmed COVID-19 who experienced stroke

Baseline characteristics	COVID-19 patients with stroke (*n*=18)
Demographics	
Type of stroke, *n* (%)	
Ischaemic	11 (61.1)
Haemorrhagic	7 (38.9)
Age (mean ± SD)	62 years ± 13.5
Sex, *n* (%)	
Male	10 (55.6)
Female	8 (44.4)
Comorbidities, *n* (%)	
None	6 (33.3)
Hypertension	10 (83.3)
Diabetes mellitus	7 (58.3)
Dyslipidemia	5 (41.7)
Obesity	5 (41.7)
Cardiovascular diseases	4 (33.3)
Neurological diseases	2 (16.7)
Malignancy	1 (8.3)
Hypothyroidism	1 (8.3)
Down’s syndrome	1 (8.3)
Smokers, *n* (%)	4 (22.2)
Clinical parameters	
Initial COVID-19 symptoms, *n* (%)^a^	
None	3 (16.6)
Respiratory symptoms^b^	14 (77.8)
Neurological symptoms^c^	1 (5.6)
ICU admission, *n* (%)	15 (83.3)
Respiratory support, *n* (%)	
None	3 (16.7)
Non-invasive ventilation	4 (22.2)
Mechanical ventilation	11 (61.1)
Stroke symptoms, *n* (%)	
Impaired consciousness	5 (27.8)
Hemiparesis	8(44.4)
Headache	2 (11.1)
Dysarthria	4 (22.2)
Aphasia	1 (5.6)
Mental confusion	2 (11.1)
Facial paresis	3 (16.7)
Coma (mydriatic eyes)	7 (38.9)
Outcomes, *n* (%)	
Death (mRS^d^6)	12 (66.7)
Discharged (3 mRS5; 2 mRS4; 1 mRS3)	6 (33.3)
Time difference between COVID-19 diagnosis and onset of stroke symptoms, median [range]	11 days (0–31)
Laboratory parameters	
Electrocardiogram (ECG), *n* (%)	
Atrial fibrillation	4 (22.2)
Sinus bradycardia	14 (77.8)
WBC [10^9^/l], median [range]	10.85 (2.57–29.3)
Platelet count [10^9^/l], median [range]	210 (78.5–375)
Haemoglobin [g/dl], median [range]	12.75 (10.1–15)
Albumin [g/l], median [range]	35.25 (24.6–37.9)
ALT [IU/l], median [range]	26 (12–88)
AST [IU/l], median [range]	36 (10–160)
LDH [IU/l], median [range]	496.5 (222–2060)
Troponin I [ng/ml], median [range]	0.031 (0.007–0.594)
INR, median [range]	1.1 (1.0–1.62)
aPTT, median [range]	36.3 (28.1–56.2)
D-dimer [µg/ml], median [range]	8.65 (0.61–18.19)
Ferritin [ng/ml], median [range]	618.2 (176.8–2963)
Procalcitonin [ng/dl], median [range]	0.215 (0.051–17.14)
CRP [mg/l], median [range]	38.15 (2.5–3.568)
Creatinine [mg/dl], median [range]	1.056 (0.64–6.33)
CPK [IU/l], median [range]	99 (2.78–754)
IL-6 [pg/ml], median [range]	35.15 (4.9–571.9)

ALT, alanine transaminase; aPTT, partial thromboplastin time; AST, aspartate aminotransferase; CPK, Creatinine phosphokinase; CRP, C-reactive protein; IL-6, interleukin-6; INR, international normalized ratio; LDH, lactate dehydrogenase; mRS, Modified Rankin Scale; WBC, white blood count.

a Initial COVID-10 symptoms are defined as typical COVID-19 symptoms that occurred before stroke onset.

b Respiratory symptoms are defined as fever, cough, shortness of breath, sore throat, fatigue.

c Neurological symptoms are defined as headache, dizziness, loss of smell and taste.

d The Modified Rankin Scale (mRS) is used to measure the degree of disability in patients who have had a stroke, as follows:

0: No symptoms at all.

1: No significant disability despite symptoms; able to carry out all usual duties and activities.

2: Slight disability; unable to carry out all previous activities, but able to look after own affairs without assistance.

3: Moderate disability; requiring some help, but able to walk without assistance.

4: Moderately severe disability; unable to walk without assistance and unable to attend to own bodily needs without assistance.

5: Severe disability; bedridden, incontinent and requiring constant nursing care and attention.

6: Dead.

**Table 2:** Neuroimaging characteristics of patients with COVID-19 and stroke.

Case	Computed tomography (CT) brain results
1	Large hypodensity in the territory of the left middle cerebral artery (MCA).
2	Hypodensity involving the left frontal, parietal and temporal lobes, in the head of the left caudate nucleus causing mild compression of the ipsilateral ventricle and 2 mm right midline shift with acute/subacute ischemic infarct in the left MCA territory.
3	Large left fronto-parietal intraparenchymal and left lateral intraventricular bleed. Right smaller parietal intraparenchymal bleed, vasogenic oedema, 9 mm midline shift to the right with narrowing of the left ventricle. Blood in 4rth and 3rd ventricle. Blunting of the interpeduncular and suprasellar cisterns due to brain oedema and trans tentorial herniation. Superior sagittal sinus suggesting thrombosis.
4	48×36 mm cortico-subcortical hypodensity in left fronto-insular lobe suggesting acute infarct.
5	Cortico-subcortical and white matter hypodensity in the left frontal lobe.
6	Faint left posterior and upper parietal cortico-subcortical dedifferentiation suggesting early infarct.
7	Cortico-subcortical hypodensities in the right occipital and frontal lobe with loss of grey-white matter.
8	Large acute hyperdense intraparenchymal bleeding in the left fronto-temporal lobe measuring 5.2×5.4 cm, dissecting bilateral lateral, 3rd, 4rth ventricles with 16 mm midline shift to the right, diffuse brain oedema, intracranial hypertension and impending herniation.
9	Intraparenchymal bleeds being multiple most prominent in the left parietal and right cerebellar lobes, largest measuring 41×32 mm, bilateral subarachnoid haemorrhage, increased intracranial pressure with impending herniation.
10	Right parieto-occipital and left temporo-occipital cortico-subcortical hypodensities. CT brain after 48 h: increase in the size of hypodensities, small linear hyperdensity in the parietal aspect of the right side infarct suggesting microbleed/cortical laminar necrosis.
11	Acute right MCA infarct. Tiny hypodense areas in the periventricular regions and basal ganglia. CT brain after 48 h: the previously seen hypodensity is more hypodense. Hypodensity is now evident in the right occipital and temporal lobes consistent with subacute infarcts.
12	Suspicion of faint hypodensity in the left insula. Tiny right periventricular hypodensity compatible with an old lacunar infarct. CT brain after 24 h: the left insular hypodense area is now consistent with an acute infarct.
13	Left cerebellar 6 cm hypodensity suggestive of acute/subacute infarct. Small left occipital hypodensity and a small medial right frontal hypodensity both suggestive of acute/subacute infarcts.
14	65×45×50 mm intraparenchymal bleed centred in the right lentiform nucleus with surrounding vasogenic oedema, compression of the right lateral and third ventricle and 15 mm midline shift to the left.
15	Large hypodensity with loss of grey white matter differentiation in the right posterior cerebral artery (PCA) territory thalamus and right posterior temporal lobe suggestive of acute PCA infarct.
16	Bilateral mild discrete gyral hyperdensity in upper parietal lobe suggesting mild subarachnoid bleed.
17	Intraventricular bleeding most prominent in the left lateral ventricle, extending to the right lateral, third and fourth ventricles with 8.7 mm right midline shift.
18	Massive bleeding in the left basal ganglia, brainstem with extension to the ventricles notably posterior horns of the lateral ventricles, third and fourth ventricles, 7 mm midline shift, and supratentorial herniation.

We also report on two COVID-19 patients with haemorrhagic stroke due to their peculiarity: Case 1 is a patient with spontaneous intraventricular haemorrhage who recovered, and Case 2 is a healthy patient who developed fatal intracerebral haemorrhage then died.

## Case 1

A 61-year-old woman, overweight, with a past medical history of type-II diabetes mellitus on metformin, hypertension not on antihypertensive drugs, dyslipidemia, not on antiplatelet or anticoagulant, was diagnosed with COVID-19 infection 6 days prior to her admission. Her initial symptoms consisted of mild fever, lethargy, and myalgia. Patient presented to the emergency department (ED) when she developed a sudden onset of decreased level of consciousness, confusion, and vomiting, with a Glasgow coma score of 11 out of 15. In the ED, patient was afebrile, had an elevated blood pressure (BP) of 210/118 mmHg and was tachycardic with a heart rate of 105 beats/min, respiratory rate 20 with SpO2 of 95% on 5l/min of oxygen through a nasal cannula. She was conscious, cooperative, obeying commands, and had no gross neurological deficit. Her initial laboratory tests including (white blood cell, CRP, procalcitonin, D-Dimer, etc) were all within normal limits. An electrocardiogram did not reveal any cardiac arrhythmia. A nasopharyngeal swab for COVID-19 PCR was positive with a cycle threshold CT value 30. The patient underwent a CT chest that showed mild bilateral ground-glass opacities affecting 30% of lung parenchyma compatible with COVID-19 pneumonia.

**Figure 3:**
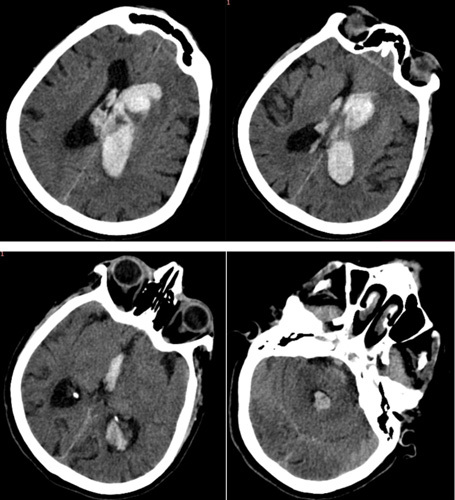
CT brain illustrating intraventricular bleeding with midline shift. CT, computed tomography.

A CT brain was performed which reveals intraventricular bleeding most prominent in the left lateral ventricle, extending to the right lateral, third and fourth ventricles with 8.7 mm right midline shift (Fig. 3).

A: Bleeding in the lateral ventricles; B: Bleeding in the third ventricle; C: Bleeding in the fourth ventricle

CT angiogram of the brain and neck did not reveal any aneurysmal dilatation or arteriovenous malformation of the cerebral vessels.

Patient was admitted to the COVID-19 ICU and started on intravenous antihypertensive medications, labetalol, to maintain systolic BP less than 160/90.

On day 5 of admission, blood tests showed a white blood cell count of 11.4×10^9^/l, elevated CRP of 83.7 mg/l, and an elevated procalcitonin of 0.223 ng/ml. Her chest X-ray showed bilateral patchy opacities. Patient was started on piperacillin-tazobactam for hospital acquired pneumonia. D-Dimer was elevated 4.8 mcg/ml, so patient started on prophylactic anticoagulation, heparin, for hypercoagulable state with close monitoring.

Her respiratory symptoms significantly improved and patient was off oxygen with SpO2 of 100% on Room Air. Her neurological status mildly improved with a Glasgow coma scale 13. Hence, she was then transferred to a regular floor, after 17 days of ICU stay, where physical therapy for her right sided weakness was initiated.

Repeated CT brain indicated further decrease in intraventricular bleeding with mild increase in the surrounding left periventricular vasogenic oedema.

Patient improved during her stay, became more conscious and cooperative, with persistent mild right sided weakness. She was discharged home after 1 month of hospital stay.

## Case 2

**Figure 4:**
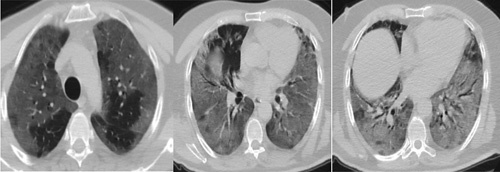
CT chest illustrating diffuse bilateral ground-glass infiltrates with a crazy-paving appearance of lungs. CT, computed tomography.

A 56-year-old male, with no significant past medical history, on no medications at home, presented to the ED with a 6-day-history of progressively worsening dyspnoea and dry cough. His vitals upon presentation were as follows: heart rate of 110/min, BP of 105/74 mmHg, temperature of 37.2 °C, respiratory rate 33, and SpO2 of 56% on room air. CT of the chest showed multiple bilateral diffuse ground-glass infiltrates with a crazy-paving appearance affecting 90% of the lung parenchyma (Fig. 4).

Patient was fully conscious cooperative, obeys to verbal stimuli, had Glasgow coma scale 15.

A nasopharyngeal swab for COVID-19 PCR was positive. Patient was started on high flow nasal canula. His oxygen requirements gradually worsened requiring intubation on day 3 of hospitalization and vasopressor support for shock on day 5, without significant change in his neurological status. D-dimer level was very elevated at 16 mcg/ml, and patient was started on therapeutic anticoagulation. Despite appropriate treatment and mechanical ventilatory support, the patient’s condition continued to deteriorate.

**Figure 5:**
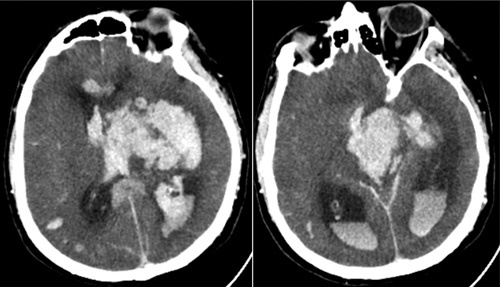
CT brain illustrating acute massive bleeding in the left basal ganglia, brainstem with extension to the ventricles. CT, computed tomography.

On day 10 of hospitalization, the patient was noted to develop bilateral dilated pupils with Richmond Agitation Sedation Scale (RASS) -5 off sedation, prompting a CT scan of the head, which showed massive bleeding in the left basal ganglia, brainstem with extension to the ventricles notably posterior horns of the lateral ventricles, third and fourth ventricles, 7 mm midline shift, and supratentorial herniation (Fig. 5).

The patient underwent a CT angiogram of head, which did not reveal any major vessel aneurysm or arteriovenous malformations. Unfortunately, the patient passed away 24 h after the onset of the massive intracerebral haemorrhage, after a hospital stay of 11 days.

## Discussion

We report the clinical and neuroimaging features of eighteen patients with COVID-19 infection who developed acute stroke, 11 ischaemic and 7 haemorrhagic.

There is still an uncertainty in the relationship between stroke and COVID-19. It is unclear whether it is causative or just coincidental. The mechanisms that have been postulated to cause neurological injury and cerebrovascular events in patients with severe COVID-19 are cytokine storm, embolic events in the background of myocarditis, and arrhythmia, hypoxia-induced ischaemia and apoptosis, thrombotic microangiopathy, coagulopathy and thrombocytopenia, or direct viral invasion^13^. Eleven of the 18 stroke patients presented in the study were found to have ischaemic type, among those patients, 45.5% has small-vessel occlusion, 36.4% of cardio-embolic origin and 18.1% from large vessel thrombosis^14,15^. These findings were different from the case series of Texas where 60% of ischaemic stroke patients had an undetermined aetiology, which could potentially be attributable to a hypercoagulable state from COVID-19 infection^8^. Other studies from Iran and New York reported an increased thrombotic state that can be associated with large-artery thrombosis^11^.

The median time to develop stroke in our case series was 12 days from the diagnosis of COVID-19 and was much longer in the critically-ill patients. In fact, many of these critically-ill patients had a delayed diagnosis of stroke based on neuroimaging findings due to a lack of clinical suspicion in presence of multisystem dysfunction and/or likely difficulty in assessing the neurological status, particularly in sedated and ventilated patients^16^.

In our study, we reported key demographic and clinical characteristics of patients who develop stroke associated with CoV-2 coronavirus infection. Some patients, specifically those with older age, a mean of 62 years old and chronic medical situations such as diabetes, hypertension, dyslipidemia, malignancy, obesity and, others are at risk to develop respiratory and neurologic symptoms, similar to other studies^17^.

Similarly to other studies^18,^ our patients with ischaemic and haemorrhagic stroke had higher markers of inflammation and coagulation such as elevated IL-6, procalcitonin, ferritin, and D-dimer, as well as more signs of COVID-19 illness severity such as ICU admission or mechanical ventilation. While elevated D-dimer levels are suggestive of hypercoagulability, they do not establish causality between CVD and COVID-19^8^. Furthermore, patients with severe COVID-19 and stroke had significant associations with greater frequencies of leukocytosis, lymphopenia, and cytolysis (measured by lactate dehydrogenase), hepatic and renal dysfunction, and deep vein thrombosis^19^. Another consideration is the role of a cytokine storm resulting in elevated levels of both IL-6 and CRP, which have been associated with an increased risk of both stroke and myocardial infarction when elevated in healthy individuals^9^.

When compared with other COVID-19 outcomes, death was the most common outcome in our case series, which is consistent with other studies^20^. The higher mortality among those with stroke may be explained by the fact that severe COVID-19 infection may have masked neurologic symptoms and led to deferral of neuroimaging evaluations, a higher prevalence of in-hospital thrombotic complications, or ineligibility for acute interventions^21^.

## Conclusion

In conclusion, ischaemic and haemorrhagic strokes can occur in COVID-19 patients and complicate the course of the infection. In our large case series of 18 patients, most of the patients had a poor outcome highlighting the need to assess all patients with severe COVID-19 infection for the risk of developing acute stroke through performing inflammatory and coagulation markers.

## Ethical approval

All procedures performed comply with the ethical standards of the national research board, IRB review was taken. Privacy and confidentiality of the patient was secured. A consent form was obtained from the patient’s family after explaining the purpose and the objective of this report.

## Consent

Written informed consent was obtained from the patient’s family for publication of this case series and any accompanying images. A copy of the written consent is available for review upon request by the Editor-in-Chief of this journal.

## Author Contribution

Z.M., G.S., M.H. and T.H. contributed to the management of the case and the conceptualization and planning of the case series. M.Z. and Z.M. contributed to its analysis. Z.M., G.S. and T.H. provided the preparation of manuscript. All authors read and approved the final manuscript.

## Conflicts of interest disclosure

The authors declare that they have no competing interests and the research is not funded by any funding agency.

## Provenance and peer review

Not commissioned, externally peer-reviewed.
